# The Treatment of Inguinal Hernia in Young Children

**Published:** 1908-11-14

**Authors:** J. Bernard Dawson

**Affiliations:** late House Surgeon to the Hospital for Sick Children, Brighton


### SPECIAL ARTICLE.

### THE TREATMENT OF INGUINAL HERNIA IN YOUNG CHILDREN.

By J. BERNARD DAWSON, B.S., F.R.C.S., late House Surgeon to the Hospital for Sick Children, Brighton.

Whilst it is no object of this paper to deal with the classification of herniæ in children, yet it is necessary for the sake of clearness to have some such basis. Primarily it is to be understood that simple reducible inguinal hernia is the condition to be treated of herein. Herniæ in children have been subdivided into several classes under several headings. For our present purposes they will be called congenital, for whether they be infantile or funicular, they are none the less congenital. Children are not the subjects of acquired herniæ.

During the past 18 months much notice has been attracted to what is known as the "Funicular Theory of Hernia," which attempts to prove that all herniæ in infants or adults are due to the persistence of a more or less patent funicular process. Whilst there is much and accumulating evidence in support of this ingenious theory, it cannot be yet accepted as proven. It is not easy to banish from our minds and text-books the classical views upon "acquired herniæ." As far as children are concerned, however, there is no dispute that hernia is due to the persistence of a funicular process. The treatment in children may occasion considerable anxiety and disappointment. It can for convenience be divided under the following headings:—

### A.—In Infancy.

The treatment from birth to two years of age should be mechanical and expectant; and conditions such as phimosis should be dealt with. Radical cure at this age is difficult, unsatisfactory, and often unnecessary. Many congenital herniæ can be cured by truss treatment, efficiently and conscientiously carried out, so that early operation is not justified until the former method has been well tried. The difficulties of operating at this stage arise from the proximity of the inguinal region to the perineum, and the difficulty of obtaining or maintaining asepsis; the strain put upon the stitches by the child crying and straining; and the frequent soiling of the dressings. Mechanical treatment consists of the applications of a truss. The best, most economical, and most efficient contrivance is usually a 2-inch flannel bandage applied as follows:—

The skin is first washed, dried, and then powdered with Fuller's earth or with equal parts of boracic acid, oxide of zinc and starch. A pad of boracic lint of suitable size is then placed over the external ring and inguinal canal, care being taken to see that the pubic spine and crest are not subjected to pressure. The bandage is then doubled in half, and the loop placed upon the lint pad. The double strip is then passed round the iliac region upon the opposite side to the hernia, and mid-way between the iliac crest and the great trochanter, the ends being then brought forward and passed through the loop.

The hernia is now reduced, maintained by digital pressure, whilst the pad is slipped back into place, and the free ends carried under the perineum and over the buttock of the affected side, to be secured to the horizontal limb of the truss.

This plan, unless the hernia be unusually large, will maintain the competence of the inguinal canal. If it fails to do so either the pressure is misdirected or is insufficient. Several such flannel trusses should be in use at the same time, so that the child may be bathed without the support being removed. After bathing, a clean dry truss is applied whilst the soiled one is washed and prepared for future use.

Great care must be taken to maintain the hernia with the finger whilst changing the bandage. The flannel bandage will be found more successful than the skein of worsted which is often recommended, but which is expensive and becomes hard and uncomfortable after washing. If the abdominal ring is very large a flannel truss will not act; in an infant in whom the conditions are not favourable for a radical cure india-rubber trusses with air or water distended pads can be tried, several patterns of which can be seen in any instrument-maker's catalogue.

During the two years of this mechanical treatment, attention must be paid to the nourishment and strengthening of the child. An ill-nourished newborn infant with congenital hernia may develop, under careful management, into a strong child free from defect, the rapid growth of the abdominal muscles shutting off the patent sac.

In the course of hospital practice the writer saw a child, aged two months, who possessed five herniæ, namely, an umbilical, two inguinal, a femoral, and a perineal. Efficient support by trusses seemed impossible and radical cures out of the question. The child was admitted to the hospital, very carefully nursed and fed, so that it grew well nourished and sturdy. With the growth of the abdominal muscles all the herniæ disappeared save the left inguinal, which was subsequently cured by truss treatment. If at the end of two years' support the hernia is still existent, the case passes into the second division.

### B.—From Two to Six Years of Age.

When a hernia has persisted from infancy the chances of cure by mechanical means become increasingly smaller month by month, and after six years of age are very remote. The question, therefore, arises at what time it is best to perform a radical operation. Undoubtedly the earlier the radical cure the better, for by delay the openings become larger and more difficult to close adequately. Again, after two years the child begins to get about and be exposed to injury, and the risk of strangulation becomes correspondingly greater.

The temperament of the child is also of some account. The easily-frightened patient, who cries at the sight of the doctor, is not a good subject for early operation, whilst the cleanliness of its habits are a factor to be considered. Given favourable conditions, however, the earlier the operation after the second birthday, the better the chances of a satisfactory result. If there be reason to delay the radical cure a truss should be worn. This should be a light spring truss of the usual pattern, but covered entirely with india-rubber.

### C.—After Six Years of Age.

If the hernia remains uncured or undiscovered until after six years, a radical cure is the best treatment. At this age the child is manageable and clean in habits. Yet he is not old enough to manage a truss himself, and may allow the hernia to descend below the pad without giving notice to his parents. He is also at an age of shyness and acute feeling which may be badly hurt by school-mates who taunt him upon his inability to join in their pastimes.

### D.—The Operation.

In an ordinary congenital hernia the surgical procedure of radical cure is simple and easy. The operation which succeeds admirably is a modification of Bassini's. If the hernia is small and the truss not unduly large, it suffices to obliterate the sac completely.

As a rule no attempt need be made to approximate the lower edge of the conjoined tendon to Poupart's ligament. The sac can be obliterated either by ligature or by torsion. If the former method be used it is a useful step to leave the ends of the ligature long. These are threaded on a curved blunt aneurysm needle, one by one, and thrust through the aponeurosis of the external oblique some ½ inch external to the site of the internal ring. Traction will then draw the peritoneal dimple away from the internal ring, which is the point of weakness in the abdominal wall. By securing the ligature ends, a plane peritoneal surface is thus brought over the internal ring.

Whether the aponeurosis of the external oblique should be divided over the inguinal canal or not, is a matter of choice. Such division gives a better view of the state of affairs and allows of scientific handling of any condition present. The divided fascia unites readily if brought into good apposition. To work through the external ring without dividing the fascia produces less disturbance of tissue, but necessitates manipulating somewhat in the dark.

One other word may be said as to the difficulty, sometimes extreme, in recognising the sac from other constituents of the spermatic cord. This delicate sac can easily be damaged and rendered unrecognisable by careless handling.

